# Functional amyloids as molecular switches: emerging roles in diverse signalling pathways

**DOI:** 10.1042/BST20250193

**Published:** 2026-08-25

**Authors:** Jessica A. Buchanan, Brayden C. Williams, Megan Steain, Margaret Sunde

**Affiliations:** 1 School of Medical Sciences, Sydney Nano and Sydney Infectious Diseases, The University of Sydney, Sydney, NSW 2006, Australia; 2 School of Medical Sciences, Charles Perkins Centre and Sydney Infectious Diseases, The University of Sydney, Sydney, NSW 2006, Australia

**Keywords:** fibrils, functional amyloid, signaling

## Abstract

Across all domains of life, functional amyloid fibrils serve structural, storage and signalling functions. In signalling pathways, assembly into the amyloid state constitutes a rapid and cooperative molecular switch that can reorganise protein function, promote multivalent interactions, and amplify downstream responses through templated self-propagation. Advances in the understanding of functional amyloids as regulators of signalling highlight their tailored structural properties and biological consequences. The nucleation-dependent polymerisation of amyloid enables sensitive control over activation thresholds, while the repetitive fibrillar architecture concentrates functional domains to enhance avidity and cooperativity. Controlled mechanisms for assembly, compartmentalisation and reversibility prevent inappropriate persistence or activity. These features underpin diverse signalling systems, including the RIPK1:RIPK3 necrosome in mammalian necroptosis, fungal prion-based cell-death pathways and prion-like regulatory systems in yeast and metazoans that modulate translation, development and cellular memory. Functional amyloids represent a versatile and evolutionarily conserved mechanism for molecular signalling and offer opportunities for the design of programmable amyloid-based switches with potential applications in therapeutics and synthetic biology.

## Introduction to functional amyloids

**Figure 1:**
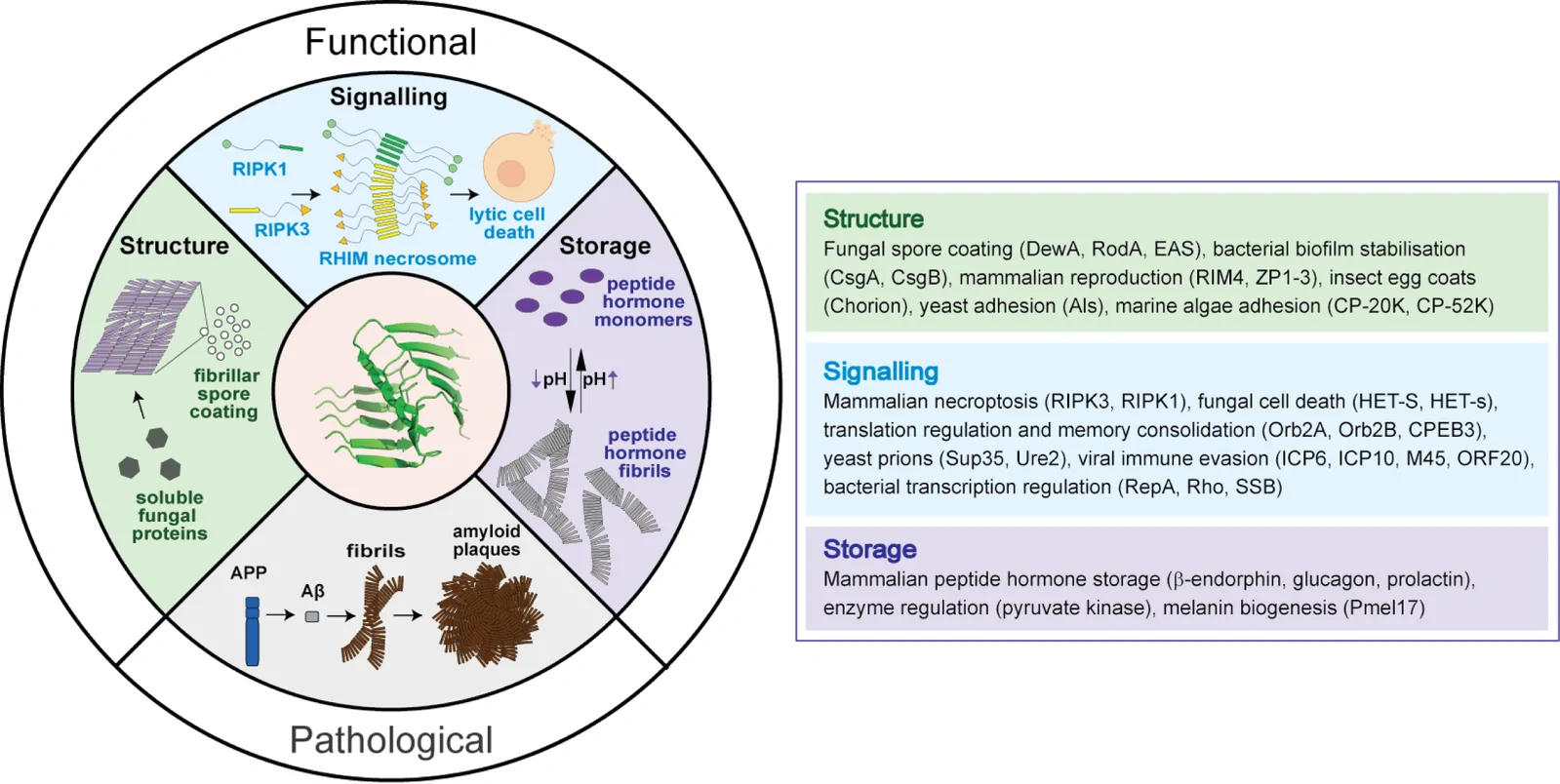
Functional and pathological amyloids All amyloids have a cross-β core structure (centre). Broadly, functional amyloids play structural, signalling or storage roles. Structure: fibrils form robust, protective protein coatings on airborne fungal spores (hydrophobin proteins); Signalling: fibrils localise functional domains which propagate molecular signals (RHIM necrosome with kinase domains); Storage: fibrils in secretory granules form a controllable polypeptide repository (β-endorphin peptide hormone). The Aβ peptide (Aβ), derived from the amyloid precursor protein (APP) forms large extracellular fibrillar plaques in AD. Key examples of functional amyloids with structural, signalling or storage roles are listed on the right.

The cross-β amyloid fibril architecture, long associated with protein misfolding and human disease [1–5] and adopted by many proteins under denaturing conditions [6–8], also forms the core of many functional biological assemblies with diverse roles across the kingdoms of life [9,10] (Figure 1). Cryo-electron microscopy has revealed how both common and unique structural features across disease-associated and functional amyloids are accommodated in two-dimensional folds which repeat along the fibril axis [11,12]. Multiple intermolecular sheets pack against each other, and the combination of inter-strand hydrogen bonds, π–π aromatic stacking, ladders of stabilising salt bridges and van der Waals forces in dry inter-sheet ‘steric zippers’, generates highly stable fibrils [13].

The functions or biological activities and properties conferred by functional amyloids include generating protective or stabilising fibrillar structures, acting as polypeptide storage depots, modulating metabolism or forming platforms which facilitate biological signalling (Figure 1). In regards to the structural function, robust amyloid fibrils provide protective protein layers on the surface of fungal spores (hydrophobin proteins) [14] and within mammalian oocytes and sperm [15], shield Gram-negative bacteria from predatory bacteria and bacteriophages [16], stabilise biofilms (bacterial curli) [17,18] and insect egg coats [19], and contribute to adhesion in marine algae and yeast [20,21]. Other functional amyloids represent polymeric storage forms, such as depots of mammalian peptide hormones (including β-endorphin) in secretory granules, where the fibril form is readily reversible in response to appropriate physiological or environmental cues [22–24], or enzymes such as pyruvate kinase which assemble into an enzymatically inactive fibrillar form under conditions of metabolic stress and are resolubilised to active and soluble forms when the stress is relieved [25,26]. The third group of functional amyloids comprises those where the conversion to the fibrillar form provides a switch or signal for downstream events and modulates activity through recruitment, colocalization or sequestration of specific proteins.

The templating nature and widespread distribution of amyloidogenic motifs across known genomes suggests that simple amyloids may have played a role in the origin of life, contributing chemical and biological activity through repetitive assembly [27]. However, although most protein sequences contain segments that are amyloidogenic [28,29], only very few will form amyloid under biologically relevant conditions [30]. Those that do contain evolved amyloidogenic sequences, associated with specific control mechanisms which reflect the activity and niche where amyloid assembly is employed [9–11,31]. Exposure of the amyloidogenic motif occurs in response to environmental conditions or a specific stimulus.

## Functional amyloids with roles in signalling: altered activity or functionality

Amyloid fibrils possess a set of emergent structural properties that make them particularly well adapted for signalling functions [27,32]. The incorporation of a soluble protein into the cross-β amyloid fibril structure typically represents a sharp, cooperative transition, effectively enabling binary switching between two states. The repetitive nature of the amyloid structure provides a mechanism for assembly of different proteins through common motifs. Once nucleated, amyloid fibrils grow by templated monomer addition, which ensures that the functional fold remains the most energetically favourable, persists and allows faithful transmission and amplification of the conformational information. The resultant gain or loss of function will depend on the impact of the structural transition which occurs upon incorporation into the fibril [32]. Perhaps uniquely for functional amyloids which are involved in signalling pathways, the molecular switching engendered by amyloid assembly can recruit, amplify or repress multiple biological activities, including activities which depend on avidity or cooperativity effects. This is because functional amyloids involve proteins with distinct functional domains adjacent to the amyloidogenic motifs.

**Figure 2:**
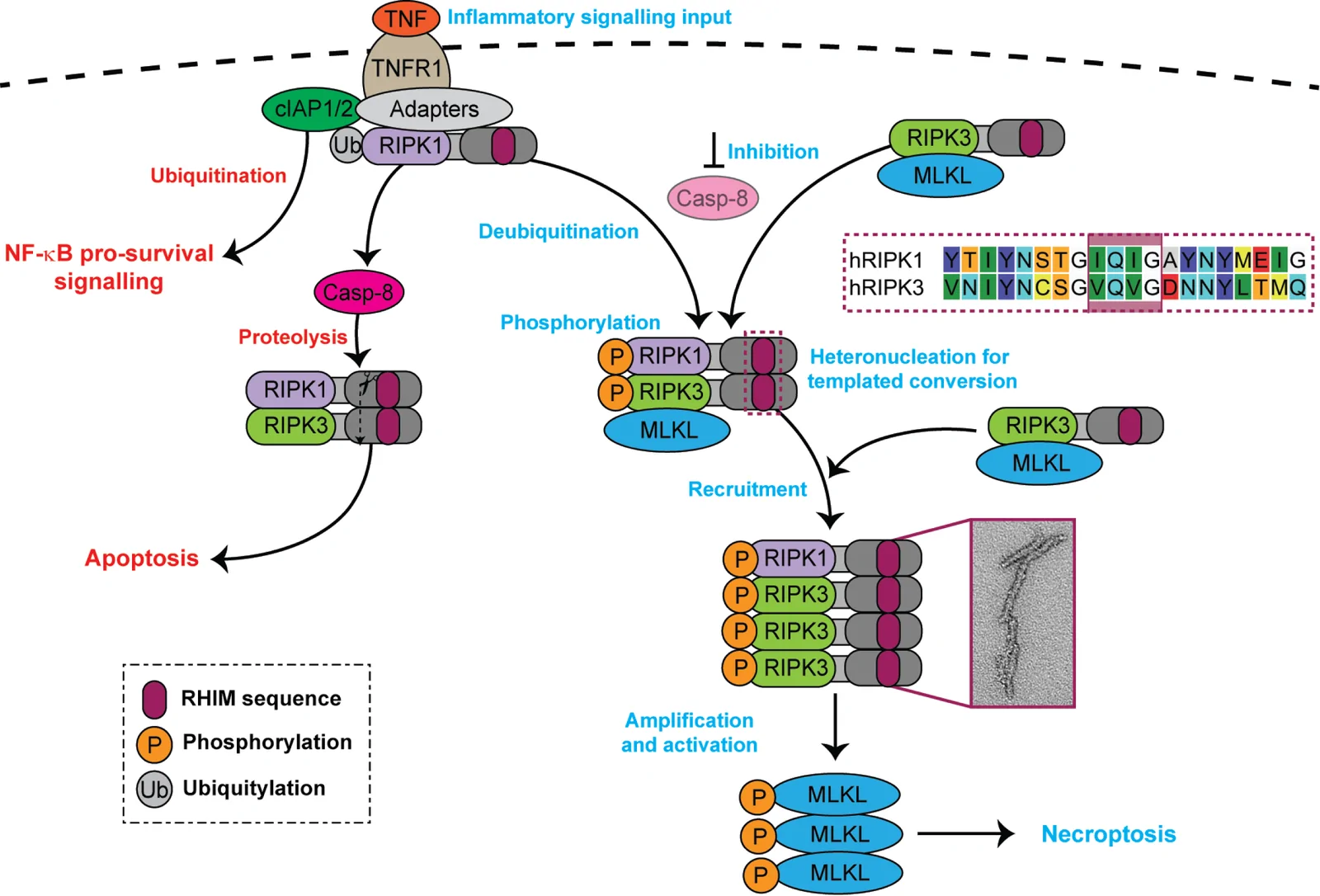
Functional amyloid signalling in necroptosis Necroptosis is triggered by TNF downstream of inflammation. Ubiquitination of RIPK1 within the TNFR1 complex acts as a negative regulator of necroptosis, as does caspase-8 (Casp-8) cleavage of RIPK1 and RIPK3, leading to NF-κB pro-survival signalling or apoptosis, respectively (anti-necroptotic signalling shown in red). RIPK1 deubiquitination and Casp-8 inhibition leads to RIPK1:RIPK3 amyloid complex formation via the RHIM (motif with core tetrad highlighted). This heteromeric structure nucleates further RIPK3 recruitment into the amyloid structure (electron micrograph of homomeric RIPK3 fibrils), resulting in amplified phosphorylation of RIPK3 and MLKL, subsequently leading to MLKL oligomerisation and lytic cell death (pro-necroptotic signalling shown in blue).

The role for functional amyloids in signalling is exemplified by the proteins which form an amyloid-structured platform for signalling in the programmed cell death pathway, necroptosis (Figure 2). This pathway occurs downstream of inflammation and the release of tumour necrosis factor (TNF). Binding of TNF to TNF receptor 1 (TNFR1) promotes the assembly of signalling complexes containing RIPK1 which regulate cell fate. While this signalling can drive pro-survival pathways, under specific conditions it is redirected towards cell death. In this context, induced conformational change in RIPK1 exposes an amyloidogenic sequence. It engages the kinase RIPK3, which contains a closely related sequence, to form a heteromeric amyloid signalling complex. The RIPK1:RIPK3 fibril structure is known as a necrosome, and it was the first mammalian functional amyloid reported with a signalling function [33].

Amyloid assembly usually occurs through a nucleation-dependent polymerisation mechanism [34]. Intermolecular assembly through amyloidogenic motifs results in the formation of an oligomeric nucleus, with subsequent recruitment of additional monomers, and fibril elongation leading to the formation of mature amyloid fibrils. The RIPK1:RIPK3 complex can be considered as the nucleus for fibril assembly as it triggers further recruitment of RIPK3 molecules and fibril elongation (Figure 2). With recruitment of additional RIPK3 molecules into the growing fibril, RIPK3 kinase domains become concentrated and localised, facilitating autophosphorylation [35]. This leads to phosphorylation of the downstream effector protein, MLKL, exposure of its executioner domain, multimerisation, membrane localisation and ultimately, lytic cell death [36,37]. RIPK1 and RIPK3 are observed to form small heteromeric structures an hour after necroptosis is induced by TNF, when caspase-8 and apoptosis are inhibited [38]. After four hours, fibril-like structures containing the two proteins can be visualised, with cell death following fibril appearance and lysis propagating inflammatory signalling to adjacent cells [38]. The motif through which RIPK1 and RIPK3 interact is known as a Rip Homotypic Interaction Motif (RHIM). RHIMs are 18–20-residue segments with an amyloidogenic character and a highly conserved core tetrad (V/I)-Q-(V/I/L/C)-G [33,39,40]. RHIMs are also present in two other mammalian proteins TRIF and ZBP1 [41]. Amyloid-structured necrosomes can be assembled consisting of TRIF, RIPK1 and RIPK3 in the TLR3/4-induced necroptosis pathway, and ZBP1, RIPK3 and RIPK1 in the Z-nucleic acid-induced necroptosis pathway [42].

Beyond their canonical role in necroptosis, RHIM-containing signalling complexes can also promote inflammatory responses independently of cell death. Ubiquitination of RHIM complexes containing ZBP1, RIPK1 and RIPK3 can facilitate activation of NF-κB and other inflammatory pathways, resulting in the production of cytokines and chemokines [43]. These necroptosis-independent functions may contribute to host defence against neurotropic viral infections. Consistent with this, RIPK3 oligomerisation with other RHIM proteins has been shown to activate antiviral and inflammatory gene transcription in neurons in response to Zika and West Nile virus infection [44]. Furthermore, inherited RIPK3 deficiency in humans has been associated with susceptibility to herpes simplex virus type 1 (HSV-1) encephalitis [45], highlighting an important role for RIPK3-mediated immunity in the central nervous system.

## Structural changes for functional consequences

A functional amyloid in the filamentous fungus *Podospora anserina*, with mechanistic similarities to the mammalian RHIMs, serves a dedicated cell-death inducing role in heterokaryon incompatibility, a form of innate immunity that prevents fusion between genetically dissimilar strains [46]. The HET-S cell death-inducing protein has two domains, an N-terminal death execution domain HeLo, with similarities to MLKL, and a C-terminal prion-forming domain containing an amyloidogenic motif with some sequence similarities to RHIMs [47]. Conversion of the C-terminal prion-forming domain to a highly ordered β-solenoid fold [48] induces a structural change in the HeLo domain, which adopts a pore-forming conformation. This occurs when the cytoplasmic contents of HET-s and HET-S strains are combined, leading to localized cell death at heterokaryon fusion sites via the HeLo domain [49]. This execution pathway can also be activated by the Nod-like receptor NWD2, which contains a similar prion-like domain which can template amyloid formation in HET-s [46,50]. Analogous signalling systems with RHIM-like sequences and effector proteins, and exhibiting prion-like activity, have been identified in bacteria which form multicellular filamentous structures (e.g. *Actinobacteria* and *Cyanobacteria*) [51]. In these systems, RHIM-like bacterial amyloid signalling sequences (BASS) are present at the C-terminus of predicted helical domains analogous to fungal HeLo domains, and at the N-terminus of Nod-like receptors. The BASS motifs have been shown to form amyloid fibrils and the prion nature of these aggregates, combined with the structural similarities between certain bacterial domains and cell death executioner proteins, suggest that these also combine as amyloid-based signalling systems for immune-related host defence and cell death [51].

**Figure 3:**
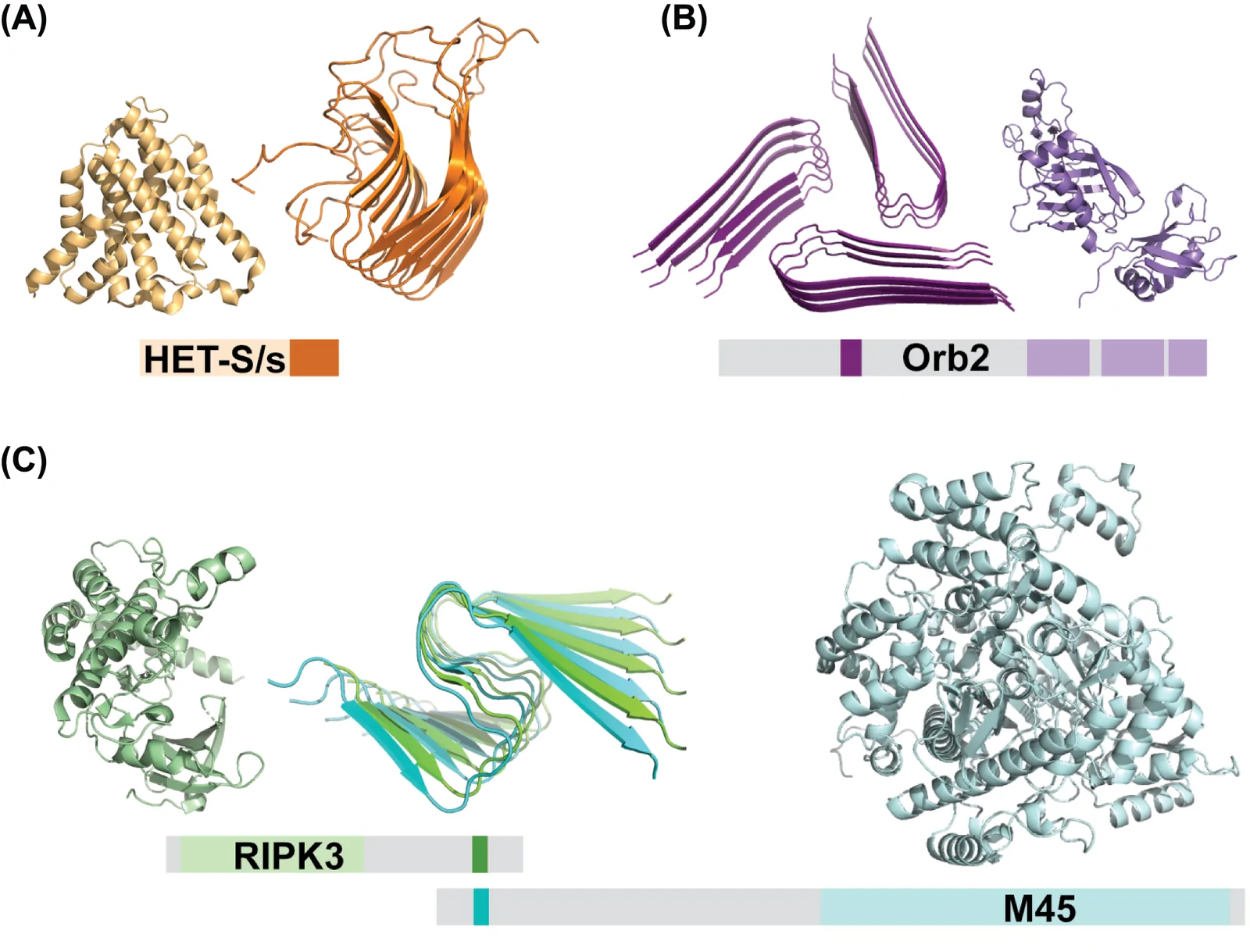
A variety of domains presented on different cross-β scaffolds (**A**) Het-S N-terminal domain (2WVO) and HET-s β-solenoid structure (2KJ3); (**B**) Orb2 multi-protofilament structure (6VPS) with RNA-recognition motifs and Zn finger domain which surround the core (AFQ9VS); (**C**) Heteromeric human:viral RHIM necrosome (12KL) presents RIPK3 kinase domain (7MX3) and ribonucleotide reductase-like domain from murine cytomegalovirus protein M45 (AFQ06A28). In representations of primary structures, amyloidogenic sequences are coloured in dark shades, associated globular domains in light shades, and disordered regions in grey.

In the RHIM-containing proteins and HET-s-related proteins, regions or domains lying outside of the amyloidogenic motifs are responsible for binding to partner macromolecules, responding to input signals and/or executing functions. The amyloid structures at the core of the fibrils have distinct architectures, S-shaped for RHIMs [39] and β-solenoid for HET-S [48], but both provide a stable scaffold for the other interacting domains on the outer surface of the fibrils (Figure 3A,C). The in-register β-sheet alignment of amyloid gives rise to proximity of functional domains, multivalency and cooperativity effects, which likely increase binding avidity and amplify the signalling cascade.

Structural reorientation into a repeating amyloid fold can also create new interfaces and functions distinct from those of the monomeric form of the protein. Functional amyloid formation is therefore a mechanism for providing multi-functionality of proteins and peptides without alternate protein production. The *Drosophila* Orb2 protein illustrates this type of transition. Orb2 is an RNA-binding protein involved in memory consolidation for long-term storage. This occurs through the formation of a heteromeric amyloid consisting of two Orb2 isoforms: the highly amyloidogenic Orb2A and highly abundant Orb2B [52,53]. On neuronal signalling, Orb2A assembles and nucleates amyloid formation of Orb2B (Figure 3B). This reorientation causes a conformational change in the RNA-binding site at the N-terminus of Orb2B, switching its function from a translation repressor to a translation activator, allowing for memory formation to occur [54]. It has also been suggested that Orb2 amyloid formation is regulated by a J-domain chaperone, which may act as a trigger for Orb2A amyloid assembly in neurons [55]. Orb2A is believed to work in conjunction with Orb2B in memory consolidation, but to have a stronger role in memory storage, rather than formation [56].

Yeast prions, where conversion to the insoluble prion amyloid form modulates function and creates diverse phenotypes by altering protein availability, have been well characterised [57,58]. The prions [PSI+] and [URE3] found in *Saccharomyces cerevisiae* correspond to the amyloid states of Sup35 and Ure2. In their soluble conformations, these proteins perform essential cellular functions, including translation termination and nitrogen catabolite repression [59,60]. Switching to the prion state can occur spontaneously or in response to environmental changes and reduces protein activity, allowing yeast populations to explore alternative phenotypic states [61]. The fibril architecture is sufficiently stable to ensure signalling complexes are preserved across successive rounds of growth across generations, or even between species [62].

Budding yeast also employs non-heritable prion-like assemblies to control cell fate and two RNA-binding proteins which function through amyloid forms have been characterised in *Saccharomyces cerevisiae*. Whi3 contains a low-complexity, prion-like domain that enables it to undergo regulated condensation in response to stimuli, forming large and inactive assemblies known as mnemons, which sequester specific mRNAs [63]. This causes long-lasting changes in cell behaviour but these assemblies cannot be passed on to daughter cells, hence the signalling is spatially confined and not heritable. In early meiosis, Rim4 assembles into amyloid-like aggregates which bind and sequester target mRNAs to repress their translation [64]. At later meiotic stages, Rim4 amyloid is actively disassembled, releasing these mRNAs for translation. Rim4 therefore regulates and coordinates development through translational control. *In vivo*, the generation and maintenance of these prions depends on molecular chaperones; hence, they represent a controllable signalling state which can regulate at a population-level, distinct from canonical intracellular pathways [65].

A few prion-like amyloid-forming domains with functional roles have been reported in bacteria. The N-terminal WHI domain in the replication protein RepA of *Pseudomonas savastanoi* [66], the transcriptional terminator Rho from *Clostridium botulinum* [67] and the *Campylobacter* single-strand DNA binding protein SSB [68]. The WHI domains within full-length RepA assemble into an amyloid-structured complex that brings together two copies of the target plasmid in a ‘handcuff assembly’ to control DNA replication initiation [66,68]. The N-terminal prion domain of the helicase Rho induces transcription termination by releasing mRNA from its DNA template and aggregation of the domain leads to loss of activity [67]. The prion-related mechanism of action of SSB remains unclear.

## Regulation of functional amyloid assembly and disassembly

Regulation of functional amyloid formation and propagation occurs through a variety of highly evolved mechanisms. Often, functional amyloid formation will depend on the presence (or absence) of a proteolytic cascade. For example, the pigment-specific protein Pmel17 undergoes proteolytic processing before forming arrays of amyloid fibrils which sequester melanin within melanosomes [69–72]. Alternately, if RIPK1 and RIPK3 are cleaved by caspase-8, they lose their ability to form into a functional necrosome structure [73].

Hetero-nucleation is a common feature of functional amyloid assembly utilised in the necroptosis pathway, in the HET-S/s system and in Orb2. Nuclei are the smallest structural units which can template fibril growth and they allow for an immediate shift into the elongation phase of assembly, where fibril growth occurs. In the context of signalling, these nuclei can act as the molecular switch for fast activation because nucleation is the rate-determining step of fibril formation. In hetero-nuclei, the constituent proteins carry identical or closely-related amyloidogenic motifs which allow co-assembly into mixed or hybrid fibrils. This may provide additional regulation of signalling, with the two amyloidogenic partner proteins under separate control mechanisms.

Molecular chaperones appear to play a significant role in the controlled formation and disassembly of functional amyloid. This is exemplified by the bacterial curli cascade, which requires a series of chaperone-mediated molecular events before biofilm formation can commence [27], and is a characteristic of yeast prions [57,58,65], where all critical stages of amyloid formation and dissociation are dependent on chaperone partners. An HSP90-CDC37 co-chaperone complex has been reported to associate with RIPK3 during necroptosis [74]. Given that functional amyloids have evolved with controlled oligomerisation as a key feature, inseparable from function, optimisation of function has likely occurred alongside the mechanisms for regulating the exposure of amyloidogenic motifs. Tight control of primary nucleation and rapid aggregation kinetics are hallmarks of functional amyloid formation [75]. These features likely reduce the number of polymorphic or non-functional species. However, the considerable diversity in sequence composition and biological activity observed across those functional amyloids which have been well characterised, and the paucity of data reflecting *in vivo* assembly conditions, limit the evaluation of general principles.

Compartmentalisation and the repetitive structure of functional amyloids are important in assembly and disassembly. In some cases, amyloid is formed and sequestered in low pH or ion-deficient environments, such as the Pmel17 amyloid in melanosomes [71], biofilm-associated protein (Bap) [76] and peptide hormones in secretory granules [77]. This ensures that formation is controlled and amyloid assemblies will dissociate on contact with the neutral, ion-rich cytosolic environment [24]. Conversely, the repeated motifs in the fibril core can provide a mechanism for reversibility. Some fibril structures are stabilised through ladders of salt bridges which run along the outer surfaces of the fibrils, or clusters of compensated charges where acidic and basic residues side chains are in close proximity within the internal folds of the fibril. Lowering of the pH and protonation of the acidic residues, or local changes in the concentration of specific ions, can lead to loss of these stabilising interactions and rapid dissociation [24]. This could contribute to the turnover of functional amyloid complexes such as necrosomes and protect the cell from inappropriate persistence of the amyloid structure following completion of its function [78,79]. Compartmentalisation may alternately ensure that control mechanisms are co-localised with amyloid proteins, e.g. specific chaperones, or provide biological surfaces for nucleation: RIPK3 has been suggested to begin amyloid formation for necroptosis signalling following contact with the cellular membrane [80].

Clearance of amyloid necrosomes has been reported to occur through chaperone activity and through autophagosome-driven degradation [81]. Heat shock protein family A member 8 (HSPA8, also referred to as HSC70 or HSP70-8) has been identified as an amyloidase that interacts with RHIM-containing proteins outside of the core tetrad to prevent nucleation, as well as to dissociate fibrillar necrosome complexes [82]. The same study demonstrated that in contrast, this chaperone was unable to completely dissociate α-synuclein fibrils, highlighting differences between functional and pathological amyloid in regard to stability and reversibility of fibrils.

The incorporation of post-translational modifications is also a significant step in the generation, persistence and reversibility of amyloid-based signalling. Ubiquitination and SUMOylation play a role in the control of amyloid assembly by increasing solubility of monomeric protein and disfavouring hydrophobic-driven amyloid assembly pathways until the appropriate requirements have been met [83]. Ubiquitination of RIPK1 and caspase-8 cleavage of RIPK1 and RIPK3 act as negative regulators preventing aberrant amyloid formation, where necroptosis signalling is not required [73,84]. Cytoplasmic polyadenylation element-binding protein 3 (CPEB3) is the human orthologue of Orb2. CPEB3 contains two prion-like domains (PRDs) separated by a low complexity domain at its N-terminus. PRD1 has been found to assemble into amyloid fibrils *in vitro* at low pH (reversible with an increase to physiological pH or a reduction in ionic strength) and has been implicated in long-term memory persistence [85]. PRD2 has been reported to influence CPEB3 localisation and RNA binding interactions [86]. Prior to assembly, CPEB3 is SUMOylated and detected at p-bodies, a possible regulatory mechanism for the control of amyloid assembly *in vivo*. On neuronal signalling, CPEB3 is deSUMOylated and is translocated to polysomes for mRNA binding and translation activation [87]. Whilst it seems likely that this system operates as a functional amyloid in neuronal cells, more extensive *in vivo* investigation is required for a definitive classification of CPEB3 as a functional amyloid involved in signalling.

Liquid–liquid phase separation (LLPS) and the formation of highly concentrated protein condensates may also play a role in functional amyloid assembly *in vivo*. RIPK3 and ZBP1 have been reported to undergo LLPS via the RNA-binding protein 2′–5′oligoadenylate synthase-like protein (OASL), which binds dsRNA but lacks enzymatic activity [88]. Upon viral infection, OASL binds viral dsRNA then recruits RIPK3 and ZBP1 to form a scaffolding condensate in which the necrosome is formed. OASL recruitment of RIPK3 may enhance the formation of RIPK3 amyloid fibrils, prolonging its activation for higher levels of MLKL phosphorylation [88]. Another case of LLPS involved in necroptosis involves Tax1-binding protein 1 (TAX1BP1)-mediated recruitment of activated RIPK3 into condensates with RING finger protein 146 (RNF146), an E3 ubiquitin ligase, facilitating the proteasomal degradation of activated RIPK3 [89]. Therefore, LLPS mediated by OASL promotes necroptosis while TAX1BP1-mediated LLPS negatively regulates necroptosis.

## Functional amyloids in immune signalling pathways across the kingdoms of life

Functional amyloids appear to be evolutionarily ancient, beneficial, and well suited to roles in innate immunity. In addition to the HET-S/s system in filamentous fungi and analogous proteins in multicellular bacteria, they are found in what is predicted to be ancestral forms in crustaceans, amphibia and invertebrates. An alternate RHIM-like motif referred to as a “cryptic RHIM” (cRHIM, VxxG) was first identified in *Drosophila* IMD (immune deficiency) signalling pathways against bacterial peptidoglycans [90]. Peptidoglycan recognition proteins (PGRPs) respond to bacterial invasion by activating the adaptor protein Imd. A cRHIM was found within multiple PGRPs (PGRP-LE, PGRP-LC) and Imd [47], and both have been reported to form functional amyloid to trigger downstream signalling for antimicrobial gene expression [90,91]. In IMD signalling, amyloid formation is required but does not cause cell death, as is the case in necroptosis signalling. This suggests a tight regulatory mechanism for the clearance of amyloid in these species, with mechanisms speculated to include autophagy and proteasomal degradation through a series of post-translational modifications [92]. This pathway was also found to be negatively regulated by Pirk, which contains a RHIM-like region and disrupts signalling by binding to the PGRPs [91]. cRHIMs have also been identified in insect antiviral pathways in the insect stimulator of interferon genes (STING) [93]. STING was found to form oligomeric species via the cRHIM in response to viral detection, whilst mutation of the cRHIM caused increased viral infection in cells.

Other metazoa have been shown to contain cRHIMs involved in innate signalling through IMD-like pathways. Shrimp contain a cRHIM within scavenger receptor class B2 (SRB2) receptors, with homologues detected across the crustacean taxa. SRB2 senses bacterial lipopolysaccharides rather than peptidoglycans, like PGRPs, but can also assemble into amyloid fibrils alongside Imd to trigger an antimicrobial response [94]. Homologues of RIPK1 and RIPK3 involved in innate immune signalling have been detected in a variety of fish, frogs, mice and chickens, all of which have conserved pro-necroptotic and pro-apoptotic functions in cells [95–97]. RIPK1 and RIPK3 have been identified as originating in Mollusca and diverging in higher phyla, suggesting that these RHIM-containing proteins may have served as the basis for the development of other, more complex necroptosis machinery (ZBP1, TRIF, MLKL) that are not present in lower phyla [98].

Viruses employ their own functional amyloids to avoid host innate immunity. To evade necroptotic cell death, some herpesviruses have co-evolved to express RHIM-containing proteins which can sequester host RHIM-containing proteins into ‘decoy’ amyloids [41,99–101]. These alternate amyloid structures are functionally beneficial to viruses as they prevent downstream cell death signalling, allowing viruses to continue propagation. Known viral RHIM-containing proteins include ICP6 and ICP10 from herpes simplex virus 1 (HSV-1) and HSV-2, respectively, ORF20 from varicella zoster virus (VZV) and M45 from murine cytomegalovirus (MCMV). Comparison of the structures of RIPK1 [35], RIPK3 [102], M45 [103], RIPK1:RIPK3 [39] and M45:RIPK3 [103] fibrils shows that the amyloid cores are similar in homomeric and heteromeric fibrils. However, the extent of the core in the viral:host hybrid core is larger than that observed in RIPK1:RIPK3 fibrils and it is stabilised by additional interactions (Figure 3C). This probably accounts for the preferential formation of M45:RIPK3 fibrils over RIPK1:RIPK3 fibrils, allowing the virus to sequester RIPK3 away from RIPK1 and prevent necrosome formation. The M45:RIPK3 heteromeric fibrils may also inhibit necroptosis signalling by separating and reorienting the RIPK3 kinase domains due to the presence of the large ribonucleotide reductase-like domain of M45, preventing autophosphorylation of RIPK3 and subsequent activation of MLKL [103].

Certain bacteria have also evolved different mechanisms to undermine host functional amyloids. Enteropathogenic *Escherichia coli* and *Shigella* produce proteases that cleave within the RHIMs of RIPK1, RIPK3, TRIF and ZBP1 to prevent hetero-nucleation and the formation of amyloid-structured necrosomes. In this way they block inflammation and necroptosis, allowing the bacteria to survive and persist [104].

## Translational outlook

The last several decades have seen improvements in the understanding of the highly tuned mechanisms by which functional amyloids execute biological roles in signalling and also the determination of the structures of functional amyloids at high-resolution. The structural information now available for these macromolecular assemblies is informing AI-based modelling programs and improving confidence in predictions of amyloid structures. As a result, there is now an opportunity to design and utilise programmable, amyloid-based switches as research tools or for therapeutic use.

There is an emerging case for the use of functional amyloid-based therapeutics. For example, a recent alternate avenue for cancer therapeutics is to target and reactivate necroptosis, which is often prevented by cancer cells through the silencing of RIPK3 [105–109]. In addition to cancerous cell death, this aims to stimulate an inflammatory response to assist host immune cell infiltration for further removal of cancer cells [110]. The delivery of a programmable necrosome, which activates on some cleavable or compartment-based trigger may therefore be desirable to restore cell death signalling. Alternately, in pathologies where necroptosis is dysregulated, as in multiple forms of ischaemic injury [111], utilising the viral mechanism of decoy amyloid formation may be favourable to prevent cell death and an extended, aberrant immune response. Engineered functional amyloid-based systems have the potential to deliver persistent non-native signals, with diverse applications depending on the nature of the conjugated domains.

Small, amyloid-disrupting peptides have been developed for over a decade, more recently utilising AI-driven design, to act as β-breakers or β-capping molecules to prevent amyloid fibril growth or oligomer maturation [112–114]. These have been considered for therapeutic purposes to prevent fibril formation in pathologies such as Alzheimer’s disease (AD) and Parkinson’s disease. Now, there is considerable potential in applying aggregation-prone regions (APRs), short sequence motifs that drive amyloid formation, for targeted aggregation and degradation of proteins for therapeutic or biotechnology purposes. Delivery of synthetic peptides which match an intrinsic APR within a protein can bind and seed its aggregation, causing selective loss of function or proteostasis collapse. This targeted protein aggregation approach has been validated across systems, including tumour models (e.g. VEGFR2 aggregation to inhibit cancer growth) [115], antibacterial therapies (killing drug-resistant bacteria or disabling resistance enzymes) [116], and to knockdown protein function selectively in plants [117].

Utilising amyloid-based therapeutics has multiple caveats, most notably the inherent risks associated with off-target seeding, and the possibility of long-term persistence with the introduction of a prion-like protein. The possibility of cross-seeding between functional and pathogenic amyloids is an area of active research and informed by evidence of cross-seeding between pathogenic amyloids [118]. Many viruses produce their own functional amyloids to avoid host immune defences and this can increase infectivity and may also contribute to neurodegeneration or other pathology [41,101,119,120]. The mechanisms involved are not yet clear but the possibility of cross-seeding involving functional amyloids cannot be ignored. Additionally, the formation of large amyloid complexes within cells may become a proteostatic burden or generate immunogenicity against these engineered complexes, causing further off-target effects. Regulatory and disassembly mechanisms will therefore be crucial in the design of amyloid-based therapeutics to prevent long-term effects and cellular damage. Highly targeted delivery will also be required for effective and specific implementation, though this too presents challenges already established for the effective delivery of biologics and peptide therapeutics, such as effective membrane permeability and susceptibility to proteasomal degradation [121].

## Perspectives

- Functional amyloids are now recognised as integral, regulated components of normal biology, where their highly ordered fibrillar structures enable diverse physiological roles ranging from structural support to molecular storage and signalling, across all domains of life.
- Functional amyloids with signalling roles are multivalent fibrillar platforms which enable sensitive control of activation thresholds, amplification of downstream responses and the integration of multiple functional outputs. The assembly and disassembly of these structures is finely tuned through mechanisms governing nucleation, localisation, reversibility and clearance, ensuring spatial and temporal control of signalling.
- As structural and mechanistic insights into the range of functional amyloids grow, new opportunities will emerge for the rational design of programmable amyloid-based signalling systems with applications in therapeutics and synthetic biology.

## Open Access

Open access for this article was enabled by the participation of the University of Sydney in an all-inclusive Read & Publish agreement with Portland Press and the Biochemical Society under an agreement with CAUL.

## Abbreviations

AD: Alzheimer’s disease
APP: amyloid precursor protein
APR: aggregation-prone region
BASS: bacterial amyloid signalling sequences
CPEB3: cytoplasmic polyadenylation element-binding protein 3
HSV-1: herpes simplex virus type 1
PRD: prion-like domain
RHIM: RIP Homotypic Interaction Motif
TNF: tumour necrosis factor
